# Iatrogenic pulmonary artery perforation associated with 5-Fr catheter manipulation during pulmonary arteriovenous malformation embolization with a vascular plug

**DOI:** 10.1016/j.radcr.2021.12.054

**Published:** 2022-01-18

**Authors:** Jihoon Hong, Sang Yub Lee, Jung Guen Cha, Jaehee Lee, Donghyeon Kim

**Affiliations:** a Department of Radiology, School of Medicine, Kyungpook National University, 130 Dongdeok-ro, Jung-gu, Daegu, Republic of Korea; b Department of Internal Medicine, School of Medicine, Kyungpook National University, 130 Dongdeok-ro, Junggu, Daegu, Republic of Korea; c Department of Radiology, Gyeongbuk Regional Rehabilitation Hospital, 120 Mirae-ro, Gyeongsan-si, Gyeongsangbuk-do, Republic of Korea

**Keywords:** PAVM, Pulmonary arteriovenous malformation, CT, Computed tomography, AVP, Amplatzer vascular plug, MVP, Microvascular plug, Pulmonary arteriovenous malformation, Pulmonary artery perforation, Hemothorax, Vascular plug

## Abstract

Vascular plugs have been increasingly used because they have lower recanalization rates than coil embolization in pulmonary arteriovenous malformation (PAVM) embolization. To deliver the vascular plug close to the PAVM, a large-diameter catheter should be advanced into the feeding pulmonary artery, which carries a risk of vascular damage. Fifty-three-year-old women was admitted to our hospital for embolization of a single PAVM. Pulmonary angiography revealed a simple PAVM with a tortuous, small feeding artery in the right middle lobe, and feeding artery negotiation was attempted using a 5-Fr headhunter-type catheter to deliver the vascular plug. However, unintentional arterial perforation occurred suddenly when the guide wire was withdrawn after the catheter was advanced to the feeding artery adjacent to the sac. Immediate embolization using a vascular plug and microcoils at the proximal site of the perforation was performed to stop both PAVM shunt flow and bleeding. To prevent such a catheter-induced complication, it is necessary to select a diagnostic catheter with appropriate stiffness and angle and to switch to a small-diameter delivery system depending on the situation.

## Introduction

Pulmonary arteriovenous malformations (PAVMs) are direct connections between the pulmonary artery and vein, and transcatheter embolization is the treatment of choice for PAVMs, with high technical success [1]. Pulmonary artery perforation associated with catheter manipulation during the procedure is a rare but life-threatening event [2]. With the use of new instruments such as vascular plugs, which have been reported to be associated with low recanalization rates, the incidence of cases in which a large-diameter catheter is required to approach the feeding artery is higher than that of conventional microcoil embolization [3,4]. Negotiating a feeder with a large-diameter catheter entails a risk of vascular damage because the access route for PAVM is typically small and complex. Herein, we report the case of a patient who developed a hemothorax due to pulmonary artery perforation associated with 5-Fr catheter manipulation during PAVM embolization. In the same session, we managed PAVM and its complications simultaneously.

## Case report

**Fig. 1:**
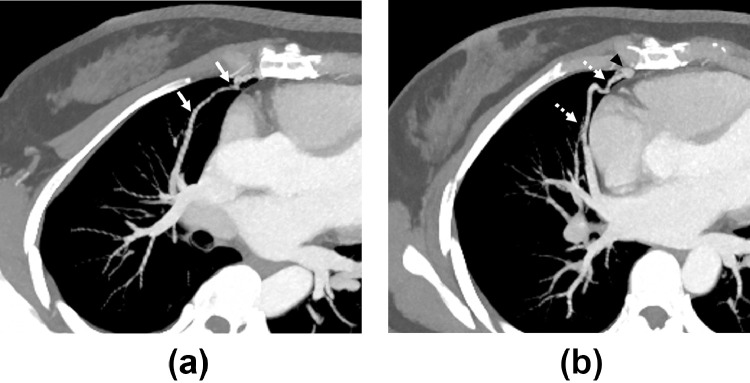
Preprocedural computed tomography images with maximum-intensity projection reconstruction (**A, B**) showing the angioarchitecture of the simple-type PAVM. The small-diameter feeding artery (arrows) with a tortuous course, venous sac (asterisk), and single draining vein (dashed arrows) are shown.

**Fig. 2:**
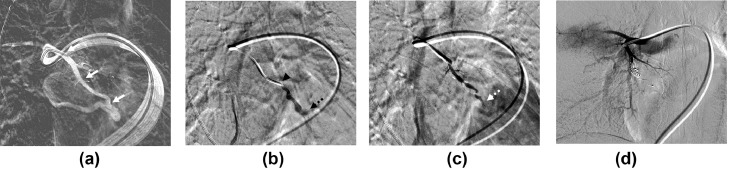
Pulmonary arteriovenous malformation (PAVM) embolization. (**A)** The roadmap image showing the angioarchitecture of the PAVM, with a small-diameter feeding artery (arrows) with a tortuous course, similar to the computed tomography finding. (**B)** Digital subtraction angiography (DSA) image showing that the 5-Fr headhunter-type catheter (arrowhead) that had access to the feeding artery adjacent to the sac (not shown) was restored to its original shape after withdrawal of the guide wire, which caused pulmonary artery perforation and contrast extravasation (dashed arrow). (**C)** DSA image obtained after deployment of a 7 mm-sized type IV vascular plug showing residual shunt flow (dashed arrow) and contrast leakage. (**D)** Completion angiography image after the additional coil embolization confirmed no residual bleeding or shunt flow.

**Fig. 3:**
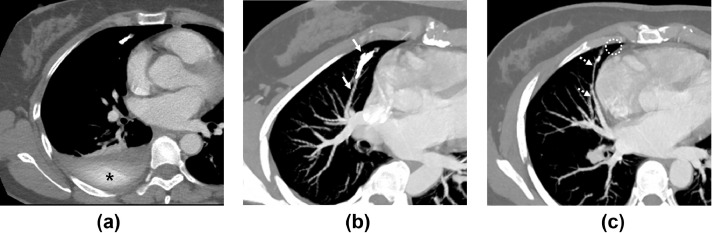
(**A)** Computed tomography (CT) image 3 d after embolization showing right hemothorax and extravasated contrast medium (asterisk). (**B, C)** Four-month follow-up CT images with maximum-intensity projection reconstruction showing resolution of all pleural effusions, occlusion of the feeding artery by the embolic devices (arrows), significant reduction in the diameter of the draining vein (dashed arrows), and almost complete disappearance of the venous sac (dashed circle).

Fifty-three-year-old women was referred to our hospital to undergo endovascular embolization for a single PAVM. Contrast-enhanced computed tomography (CT) revealed a simple PAVM with a single 3 mm-diameter feeding artery in the right middle lobe (Fig. 1). Feeding artery embolization was planned using a type IV vascular plug (Amplatzer Vascular Plug [AVP] IV; Abbott, Plymouth, MN). After common femoral venous access, angiography was performed by selecting the right pulmonary artery using an 80 cm 6-Fr guiding catheter (Flexor Shuttle Guiding Sheath; Cook Medical, Bloomington, IN) and a 125 cm 5-Fr hydrophilic catheter (Impress Headhunter 1; Merit Medical, South Jordan, UT). Similar to the CT finding, a simple PAVM arose at the medial segmental branch of the right middle lobar artery with a tortuous course (Fig. 2A). After careful negotiation, the distal feeding artery adjacent to the sac was successfully selected using a 5-Fr hydrophilic catheter and 0.035-in guide wire. However, after the guide wire was withdrawn gently, the original shape of 5-Fr catheter was restored and the angled catheter tip perforated the adjacent pulmonary artery. Contrast extravasation was observed on subsequent angiography (Fig. 2B). First, the prepared 7 mm AVP was deployed proximal to the perforation site; however, the residual shunt flow and extravasation persisted (Fig. 2C). Additional embolization was performed using 7 mm and 6 mm detachable coils (Concerto; Medtronic, Minneapolis, MN). Completion angiography confirmed that the shunt flow and contrast leakage had disappeared (Fig. 2D). After 3 days, CT revealed a right hemothorax with basal lung atelectasis (Fig. 3A). After 4 days, right pleural effusion was slightly increased, and a 10.2-Fr percutaneous drainage catheter was inserted. CT performed at the 6-month follow-up revealed that the PAVM was well occluded. The venous sac and draining vein were markedly reduced, the right hemothorax was completely resolved, and no sequelae remained except for a mild subsegmental atelectasis (Fig. 3B and C).

## Discussion

PAVM rupture during embolization is a rare and devastating complication that has been reported in only a few studies [2,5]. Because the pulmonary artery has a complex branching pattern and the PAVM is dilated by high blood flow, the risk of vascular injury may increase when negotiating a small tortuous feeding artery using a large-diameter catheter. Recently, the use of new embolic materials for PAVM embolization, including AVPs and microvascular plugs (MVPs), has increased. These have lower recanalization rates than those used in coil embolization [3,4]. In addition, embolization using vascular plugs is relatively simple and enables single-device occlusion. However, the use of these devices requires larger delivery systems than those used in microcoil embolization. For example, all type IV AVPs or ≥7 mm-sized MVPs require a 4-5 Fr diagnostic catheter for delivery. For a higher success rate in terms of recanalization, the embolic material-to-sac distance should be minimized [6]. To achieve these conditions, inevitably, the large-diameter delivery catheter should be placed juxta-proximal to the venous sac.

In the present case, two possible causes of the PAVM perforation can be postulated. One is the angioarchitecture of the PAVM. The feeding artery was small in diameter (3 mm) and had a tortuous course. In addition, as the feeding artery originated from the right middle lobe medial segmental pulmonary artery, it had a more complex U-shaped proximal course. Another cause is the attempt to approach the distal feeding artery using an inappropriately angled 5-Fr headhunter-type catheter. This catheter was advanced as carefully as possible just before the venous sac, and the guide wire was also gently removed. However, it is presumed that the angled catheter tip perforated the adjacent pulmonary artery as the proximal angle of the headhunter catheter was restored to its original shape.

To prevent significant complications during PAVM embolization, as in the present case, several factors should be considered. First, a smaller diameter delivery system should be used, especially for small and tortuous feeding arteries. Among the vascular plugs, small-sized MVPs can be delivered using a microcatheter system. However, MVPs were not available in our country at that time. Second, if the use of a 4- or 5-Fr catheter is unavoidable, the use of a soft and tip-angled catheter, which is less stiff than multi-angled catheters, should be considered. Lastly, careful manipulation of the guide wire is important. A 0.035-in hydrophilic wire can cause perforations as a result of its stiffness. We recommend using a triaxial system to access small and tortuous distal feeding arteries. For example, after a distal feeding artery is selected with a microcatheter system, a 5-Fr catheter can be inserted over the microcatheter and wire.

In the case of life-threatening hemothorax caused by spontaneous PAVM rupture, emergent embolotherapy is generally performed, which has a high success rate [7]. Similarly, when unintentional pulmonary artery perforation occurs, vascular plug and/or coil embolization should be performed proximal to the rupture site to preserve the branches to the adjacent normal parenchyma as much as possible. As the pulmonary artery perforation occurred just before the targeted PAVM sac in the present case, both the PAVM and its complications could be treated in the same session. It is helpful to insert a percutaneous drainage catheter for the accompanying hemothorax. All drainage should be performed after embolization as intrathoracic decompression before embolization may worsen bleeding [8]. Thoracotomy may be performed to prevent fibrothorax or empyema depending on the response to the initial drainage [9].

We report a rare case of pulmonary artery perforation that caused a hemothorax associated with 5-Fr catheter manipulation during PAVM embolization. To prevent this life-threatening complication, it is essential to select a catheter of appropriate stiffness and angle according to the size and course of the feeding artery, and to switch to a smaller delivery system if necessary.

## Patient consent

Written informed consent was obtained from the patient for publication of this case report and any accompanying images.

## Competing interests

The authors declare that they have no conflict of interest.
